# An Inquiry into the Pathology and Treatment of Yellow Fever, as It Prevails at New Orleans at This Time, August, 1829

**Published:** 1829

**Authors:** Lyman Cronkrite


Art. IX.—*An Inquiry into the Pathology and Treatment of Yellow Fever, as it prevails at New Orleans at this time, August*, 1829. By Dr. Lyman Cronkrite.

### Preliminary Remarks.

The nerves form several distinct systems entirely different in function, and in some measure independent of each other; in as much as each system performs its function separately and without the aid of the other—yet bound together as a whole.

Each nerve or nervous filament, has a separate origin in the brain, and proceeds to its place of destination for the purpose of performing one office and no more. Nor is one nerve or nervous filament capable of performing the office of another. Thus the optic nerve is capable of performing only the office of vision. If irritated it conveys no sense of pain, but only an impression of light. The auditory nerve cannot perform the office of seeing, tasting, feeling, nor smelling; but only that of hearing. Nor can the nerves which are distributed in the skin for the purpose of common sensation, perform any other function of the human system. Thus they have each a separate function to superintend, and are incapable of doing more.

If a part is to perform more than one function, nerves of various kinds, corresponding to the number of functions to be performed, send branches or filaments which unite in the form of a plexus, and then go to supply the part, as in the abdomen and thorax.

Anatomical investigations have led to the identification of the nerves, which perform the functions of seeing, hearing, tasting, and smelling; the nerves of common sensation, and voluntary and respiratory motion. But there are others of no less importance, whose existence is only known by the office they perform. These are the nerves of involuntary motion, secretion, &c. These may have a separate origin in the brain and course through the body, or they may arise envelop-ed in the sheaths of nerves of other functions, and without separating proceed to their place of destination, giving a nerve the appearance of supplying several functions. But that they are distinct filaments, and have a separate origin in the brain is sufficiently evident from the fact, that they perform separate functions, and further from the fact, that the functions are distinct and not dependent upon each other. Thus voluntary motion may cease in a part in which sensation is perfect, or sensation may cease while voluntary motion continues—or sensation and voluntary motion both may cease, while the involuntary actions of the circulation and secretion still continue. So may secretion be increased, depraved, diminished or suppressed while the action of the blood vessels continue.

If a nerve of voluntary motion be irritated, pain is not the consequence, but motion in the muscles to which it is distributed; or if it be divided, motion ceases, and the muscles are no longer susceptible of being excited into action.

If a nerve of sensation be irritated at its origin, or any where along its course through the body, the pain is not at the point touched or irritated, but in the part to which it is distributed; and if it be divided, the part to which it is distributed loses its sensibility.

If a nerve of secretion be irritated, secretion may be increased or depraved, the same as if the cause was applied to the secretory organ itself; or if the nerve be obstructed by congestion, or other causes, secretion may be suspended.

It is the same with all the nerves of the human system. If any of them be irritated, their functions may be increased or deranged, or if obstructed may cease altogether.

These facts are interesting in a pathological point of view. For if it shall appear, that the functions of various parts were deranged or suspended during life, and if upon examination after death it shall also appear, that the nerves which supplied those parts are diseased, we must conclude that such suspension or derangement was owing to the diseased state of the nerves.

Pathologists have divided diseases into classes, and have applied to one of them the title "Neuroses," because they have considered that there are diseases, to which they may especially give the title "nervous." But it is evidently a distinction without a difference. All diseases are nervous. That is, those morbid actions—that derangement of function—which constitutes disease depends upon the state of the nerves. As the nerves are the only agents which carry on *all* the functions in that perfect manner which constitutes health, so they only can carry them on in that imperfect or deranged manner which constitutes diseases. Deprive a part of its nerves and it is not susceptible of any action healthy or diseased. Motion, nutrition, secretion &c. cease, and death sooner or later ensues. That morbid strength and action in the sanguiferous system, which is observed in fever, depends as much on nervous influence, as that morbid strength of the muscular system does, which is observed in tetanus; and that debility of the secretory organs, and of the blood vessels, which manifests itself in fever, depends as much on the state of the nerves, as that debility of the muscular system which is observed in apoplexy. In short all action whether diseased or not, depends upon the nerves; and each morbid action depends upon the nerves as much in one disease as another. If any class of diseases are to be denominated nervous, fevers certainly have the best claim to the appellation, for in them the nerves exhibit most signs of disease.

*Of the remote and exciting causes of Fever.*—An opinion has long prevailed with the faculty, that the remote cause of fever possesses the power of directly "impairing the energy of the brain and nervous system," or in other words that it has a sedative influence. They have been led to adopt this opinion, by observing the weakness of the pulse, the coldness of the surface, the lassitude, sense of fatigue and debility, the diminished secretion, and other symptoms which usher in its first stage. But it may well be questioned, whether this debility be real, or whether it is only apparent. Whether the "energy of the brain and nervous system" is really "impaired" or whether some cause has thrown the circulation off its balance, produced under determinations and congestions upon the brain and nervous system, deranged or obstructed their functions and thereby caused the phenomena of fever.

The principal exciting causes are exposure to wet and cold, intemperance in eating and drinking, violent exercise in the hot sun, great fatigue, and excitement of the passions. These shall be considered separately.

*Exposure to Cold.*—A regular state of the functions of the skin, is indispensably necessary to the preservation of health. It is this function which tends to preserve the heat of the body at the same degree in all temperatures. If a man is exposed to a shower of rain or to chilling dews and night air, next day he finds his perspiration obstructed. The heat accumulates in his system in consequence of its natural outlet being obstructed. The pulse becomes quick, headach, lassitude, sense of fatigue and debility, redness of the eyes, &c. indicate a tendency to a disturbed equilibrium of the circulation and a determination to internal parts.

*Intemperance in Eating and Drinking.*—These causes have also a strong tendency to disturb the balance of the circulation, and produce determinations upon the brain and other internal parts, as the numerous cases of apoplexy, caused by them bear ample testimony. After a debauch, the deluded votary of *pleasure*, feels a slight headach, quickness of the pulse, thirst, heat of the skin, redness of the eyes, a fured tongue and other symptoms which indicate a tendency to a broken balance of the circulation, and which in a *yellow fever atmosphere*, are not unfrequently the precursors of disease.

*Violent Exercise in the hot Sun.*—This has also a strong tendency to destroy the balance of the circulation, and cause undue determinations upon the brain and nervous system. It is usually combined with others which increase its force. A man, with obstructed perspiration perhaps from the last nights exposure after taking "something to drive out the heat" goes to work in the hot sun. The circulation is hurried by the stimulus of the exercise, and the heat which accumulates in the system in consequence of obstructed perspiration. To this is added the stimulus of the heat of the sun, and perhaps of a full stomach. The pulse becomes quick, the eyes red, the face flushed or pale, the head pained, or giddy, nausea, lassitude and sense of debility, loss of strength in the lower extremities or of the whole system, faintness, dimness of sight and other symptoms occur, which indicate great determination upon the brain and usher in yellow fever; or if the determination to the brain be still greater, the patient dies suddenly with symptoms of violent congestive fever, or apoplexy.

*Excessive Excitement of the Passions.*—Fear in its varieties is probably the strongest of the depressing passions. If a person under the influence of fear be examined, it will be found, that the pulse is small and frequent. From this circumstance we judge, that less blood circulates through the radial arteries, than in other conditions of the body; and from analogy, that less blood in a given time goes through all the extreme vessels. The countenance also becomes pale, the skin constricted, and the blood accumulates about the heart and other internal parts. The heart labours with convulsive throbs to relieve itself of the accumulation, the chest heaves with laborious effort. This state of things must necessarily; and from the organization of the system, soon produce similar accumulations and determinations upon the brain, and not unfrequently a temporary delerium arises. This is fear in its most frantic excess, but in milder cases, it acts in the same manner only in a less degree. Here is a powerful cause for breaking the balance of the circulation, and may thus prove the exciting cause of fever.

Anger is one of the strongest of the exciting passions, and when in excess no one will pretend to deny, that it produces determinations upon the brain. The eyes become red, the countenance flushed, the expression will be hurried, and not unfrequently apoplexy ensues. Here again is a loss of equilibrium in the circulation, and why may it not be the first of those deranged actions which constitute disease.

I can see nothing in the nature of any of these causes, which should render them capable of co-operating with a sedative, in "impairing the energy of the brain and nervous system;" on the contrary, they are all, either directly or indirectly stimulating, and have a strong tendency to break the balance of the circulation, to produce congestions and determinations upon the brain and nervous system, and obstruct or derange their functions.

If miasma possesses the qualities of a sedative or narcotic, it is different from all other substances of that class in one important particular. For a person may breathe it for months, nay, years, with impunity, provided he meet with no exciting cause. Opium, digitalis, and all the numerous substances of the class of narcotics, act immediately after having been taken into the system. But miasma alone, as if forgetful of the attributes of its nature, and ignorant of its power, lies quiet in the system, until an exciting cause rouses it into action. It then goes about to "impair the energy of the brain and nervous system," and pursues its work of debility until the *vis medicatrix naturæ* becomes alarmed, and with the resolution and strength of a Hercules attacks and kicks it out of doors; or, unequal to the task, falls itself in the contest!

*Ratio Symptomatum.*—I will not stop to prosecute the needless enquiry, whether miasma is introduced into the system by way of the stomach or lungs; or whether it is absorbed by the skin. It is sufficient to know, (and the fact is self evident,) that it comes in contact with nerves which are susceptible of its influence, and which convey the impression to the brain. The system being under its influence, there is a tendency to a loss of balance in the circulation and excitability, and determinations, upon the brain and nervous system evinced by a slight disturbance of the various functions of the body, such as depraved appetite, irregularity of the secretions from the liver, slight headach and giddiness, furred tongue, restlessness, disturbed sleep, heat of the skin, sense of lassitude and debility, irregular state of the bowels and of the functions of the skin, &c.

These symptoms may continue a long time without causing much inconvenience to the patient, unless some cause sufficiently powerful, throws the circulation completely off its balance. Great determinations to the brain and nervous system then take place, obstructing or deranging their functions. The nerves of sensation exhibit signs of congestion or obstruction, by the torpor, dullness of perception, insensibility to pain &c. which prevail in every part of the body; or of increased excitement, in the pain of the head, back, stomach and extremities, "aching of the bones," universal soreness, restlessness and intolerance of light and sound.

The nerves of voluntary motion, exhibit signs of congestion or obstruction, by the weakness or paralysis of the lower extremities, the universal sense of debility and prostration of strength; or, of irritation, in the spasms and convulsions which in some instances usher in the disease, but more frequently arise during its progress, or at its termination.

The nerves of involuntary motion exhibit signs of congestion or obstruction, in the feebleness of the pulse and diminished re-action; or of irritation, by the increased force and frequency of the pulse, and the general increased re-action which prevail in the arterial system.

The nerves of secretion, exhibit signs of congestion or obstruction, by the torpor which prevails generally throughout the glandular system; or of irritation, by the profuse but depraved secretions which sometimes take place from the stomach, bowels, liver, skin, &c.

Should the question be asked, why miasma causes determinations to the brain and nervous system, I would answer it by asking another. How do those medicines which are called emenagogues produce a determination to the uterus, or those called diuretics, to the kidneys? External agents have various qualities, and produce specific and various effects upon the human system. Emenagogues produce a determination to the uterus, diuretics to the kidneys, and miasma to the brain and nervous system.

Determinations to the brain and nervous system having taken place, and deranged or obstructed their functions, besides the diseased action or torpor, which prevail in every part of the system, there is a torpor in the portal circle, which causes blood to accumulate there. The vessels of the stomach, intestines, liver and spleen, and all the abdominal viscera become turgid with blood. This, while it removes a large portion of blood from active circulation, and thereby prevents immediate destruction to other important organs, serves also, in turn, to keep up the torpor, to prevent secretion, and indirectly to maintain the congestion of the brain and nervous system. In the progress of the disease, reaction becomes established. Whether from suppressed secretions, accumulated excitability, an effort of the vis medicatrix naturœ, or all combined, it is perhaps unnecessary to enquire.— The blood now circulates with greater velocity, and passes more frequently through the lungs, is more completely decarbonized and consequently receives a greater quantity of caloric; which, in consequence of the torpor of the cutaneous exhalents, which in health serve to regulate the temperature of the body, the heat accumulates in unusual quantities. This acts in unison with the natural efforts of the system in increasing the violence of reaction. The *vis a tergo* of the circulation being increased, the portal circle and other congested parts become partially relieved, and the blood goes into active circulation in larger quantities, and passes more freely and copiously to the surface. Reaction, ultimately runs itself down. The brain and nervous system having sustained serious injury the various functions are carried on in an imperfect or disorderly manner. Depraved secretions take place from the stomach, intestines, liver and skin. From the surface, exudes a clammy moisture which soon putrefies. The stomach secretes an acrid matter, which corrodes and inflames its surface. The liver secretes an acrid fluid different from healthy bile.

The nerves of sensibility evince the injury they have sustained, by the insensibility which pervades every part of the system.

The nerves of voluntary motion evince the injury sustained, by the prostration of muscular strength, or by morbid and irregular muscular contraction.

The nerves of involuntary motion manifest the injury they have sustained, by the feebleness of the pulse and weakness of reaction generally; while those of secretion exhibit the same, by the paucity or depravity of the secretions.

In short, the brain and nervous system in general show the degree of injury they have suffered, by their inability further to carry on the functions of life, and the patient sinks in death.

### SYMPTOMS.

*First Stage.*—The patient is attacked suddenly with pain across the forehead, back, stomach and extremities, sickness, redness of the eyes, prostration of strength, weakness of the lower extremities, giddiness in the head, torpor of the bowels, chilliness, small frequent pulse, load at the pit of the stomach, tightness across the chest, laborious breathing, oppression at the precordia, tenderness in the epigastrum, lassitude and sense of debility, skin dry, suppressed secretions, urine sometimes suppressed. Occasionally the patient vomits bile, but more commonly only the ordinary contents of the stomach.

*Second Stage.*—The pain in the head now becomes more intense, so does the pain in the back and extremities. There is a universal soreness of the flesh and "aching of the bones." The back feels as if it was "broken." There is an acrid, burning sensation in the stomach, pain and great tenderness in the epigastric region, redness of the eyes, intolerance of light, great heat of the skin, which is usually dry, but sometimes deluged in moisture; pulse full and strong, vomiting increased, bowels still torpid, increased tightness across the chest, laborious respiration, head giddy, sense of debility and lassitude increased, tongue red, and covered with a white or yellow fur, weakness of the lower extremities, in some instances amounting almost to paralysis, countenance flushed and the expression hurried, thirst, great restlessness, watchfulness and delirium, depraved or suppressed secretions, stools watery.

*Third Stage.*—Without any symptom which indicates a favorable crisis in the disease, the patient becomes easy, "feels better," calls for food, and imagines himself recovering.—The pains gradually subside, the skin cools, but continues dry, has a soft uncteous feel, and becomes yellow; the tongue continues red, becomes dry and covered with a brown or yellow fur, the stomach at the commencement of this stage is comparatively quiet, but becomes again more irritable, pain in that organ at first nearly subsides, but comes on again, the burning acrid sensation arises now, if it had not before, bowels more easily moved; stools watery; extremities cold; breast sometimes hot; countenance hurried and anxious, slight pain and giddiness in the head; heaving of the chest; secretions still obstinately suppressed; great restlessness; black vomit; convulsions and death.

In some instances nearly every symptom of disease subsides. The pulse and skin become natural, the patient gets up, walks about his room, calls for food, and imagines himself well. This state continues from 6 to 24 hours, when in the midst of apparent security, he is seized with restlessness, black vomit, delirium, convulsions or stupor, and expires. Frequently the skin does not become yellow until after death.

Sometimes a chilliness continues during the whole progress of the disease.

*Post mortem appearances.*—The brain exhibits signs of congestion. The ventricles contain a large quantity of yellow serum. The vessels are turgid in every part of the organ, but more particularly so in the cerebellum. About the origin of the nerves there is found a network of turgid vessels. The same is observed in the spinal marrow.

The ganglionic system of nerves, as discovered by Dr. Cartwright, exhibit signs of congestion and inflammation. The stomach signs of inflammation, corrosion, and disorganization, and usually, contains the matter of black vomit. The liver is sometimes nearly natural, at others patches of inflammation are observed; the gall bladder sometimes contains a quantity of dark ropy matter, resembling vitiated bile; at others it is found empty, secretion having been entirely suppressed. In one instance, pus was found about the knee joint, in the cavity of the hip joint, and in the spermatic chord. The vessels of all the abdominal viscera turgid with blood.

In short, the appearances observed on dissection are abundantly sufficient to account for the phenomena of the disease, and the fatal termination.—For it is not to be supposed, that in a disease, in which all the functions of the body are deranged, any one part will exhibit diseased appearances to the same extent, as in those affections in which only one part is diseased, or one or two functions deranged. A disease of the whole system never falls with the same violence on any particular part, as local diseases do. Add to this, that the intervention of the third stage, between the second stage and death, gives an opportunity for morbid accumulations in some measure to subside, so that upon examination after death, diseased appearances are not observed to the same extent, as if death had happened during the first or second stage.

### *Individual symptoms separately considered, and their connexion with the Pathology of the Disease, pointed out.*

The brain and nervous system are the parts primarily affected, and the rest of the system consecutively.

Among the earliest symptoms of the disease, are the *pain and giddiness in the head.* They evidently arise from too great a determination to the brain. The pain is across the forehead, and is accompanied with a sense of fullness and heaviness above the eyes, which are red, feel full and protruded; there is pain in the eyes, intolerance of light, dilated or very small and contracted pupil. If any further evidence were necessary to prove that the brain suffers, dissection furnishes it, for its vessels are found turgid after death. Throughout the convolutions of the piamater they are gorged with blood, and the ventricles are filled with serum.

As the patient feels a *pain in the back, loins, and extremities*; and "aching of the bones," is it to be supposed, that determinations to these parts have already taken place? certainly not.—The pains are different from those which take place in inflammations. There is rather an uneasiness or aching.—The pains are not constant, but remit, intermit, and wander about. They probably arise from determinations to the brain, and nervous system; perhaps some affection of the nerves, either at their origin, or some where along their course through the body; for if a nerve of sensibility be injured at its origin, or any where along its course through the body, the pain is not at the point irritated or injured, but the part to which it is distributed. Now all the nerves are found diseased at their origin, and amongst the rest the nerves of sensibility. Wandering pains in the extremities, and various parts of the body, are frequently symptomatic of affections of the brain.

The pain in the back extends to the stomach, and is attended with a sense of tightness across the chest. This sensation corresponds with the seat of the diaphragm. The pain in the stomach is not constant, but intermits as if spasmodic. Indeed, patients have compared it to the painful sensation which arises from eating *choak cherries*, or green fruit; and which evidently arises from a spasmodic affection of the stomach and œsophagus. There is also a *sickness of the stomach*, which usually yields to no remedy. It is mitigated by some, but subsides entirely, only with the disease. These phenomena appear to be symptomatic of an affection of the brain, and nervous system. Vomiting is usually a symptom of compression of the brain, and the pain and stricture may arise from under determinations to the brain, and origin of the motor nerves and nerves of sensibility, for they are found diseased after death. Although I suppose the stomach to be affected sympathetically at first, I do not doubt its becoming the seat of a great deal of disease, during the progress of the fever.

The *torpor of the bowels*, is altogether different from that costiveness which arises during health, or in diseases of a milder character, and which in some measure depends upon a deficiency of secretion. It seems to be more nearly allied to palsy. It is a symptom of concussion and compression of the brain; and, in Yellow Fever, may arise either from a great determination to that organ, or inflammation or congestion of the ganglionic system of nerves, obstructing their functions. Cathartics do not act readily. Bloodletting, by relieving the brain and nervous system, facilitates the operation of purgatives. Those cases in which, instead of torpor of the bowels, there is a dysenteric purging, are usually milder, exhibit less cerebral affection, and yield more readily than those in which there is a torpor of the bowels. All the nerves which supply the intestines, are found much diseased after death.

*Weakness of the lower extremities.*—Immediately after the pain in the head and back, the patient feels a loss of strength in the lower extremities, and frequently either falls down, or finds it necessary to sit down. In milder cases, the patient only complains of weakness of the legs, and stumbles when he attempts to walk; in others, there is a paralysis. Is argument necessary to prove that this symptom is owing to disease of the motor nerves of the lower extremities? If so, a very good one is found, the turgid state of the vessels of the spinal marrow, and at the origin of all the nerves which go to the lower extremities.

*Suppression of Urine.*—The kidneys are supplied with nerves, whose office it is to superintend the function of the secretion of urine. These nerves have their origin in the brain. Notwithstanding any connection they may have with other nerves, or however intricate may be the connection of their filaments with those of the nerves of other functions, their *ultimate* origin is in the brain, and so long as they continue in a state of health, the secretion of urine will continue; but when they become diseased, either at their origin, or any where along their course through the body, sufficiently to obstruct their functions, the secretion of urine must be suspended. I will not enter into the controversy, whether the nerves are organs which vibrate, or whether they carry a fluid. It is sufficient for my purpose to know, that they are the organs of communication between the brain and distant parts, and that they are the organs of sensation, motion, secretion, &c.

There is a disease which occurs unconnected with any febrile affection, and which consists in a suppression of urine. Not only none is evacuated from the bladder, but none is secreted by the kidnies. The patient dies with cerebral affection distinctly marked. Suppression of urine is an attendant on the most malignant form of the disease, and is usually a fatal symptom. In every case in which I observed it, the cerebral affection was very considerable. I observed it in several instances, it occurred in one instance among my own patients. It was in a man of strong robust constitution, and full habit, and was attended with great intolerance of light and sound, constant pain in the head, and great tenderness when the head was moved suddenly, redness of the eyes, heat about the head, and throbbing of the temporal arteries.—The urine in this case was suppressed 24 hours before the attack.—The patient was freely bled and purged, and within 18 or 20 hours from the attack, urine began to be secreted. It became suppressed again, however, and the patient died with the black vomit.

*Suppression of the Secretions of the Skin and Liver.*—These like suppression of urine, are undoubtedly owing to diseases and obstructions of the nerves of secretion. They are most complete and obstinate in those cases, in which evidence of such obstruction occur.—In such cases they usually resist the action of sudorifics and cathartics, and when the ordinary means have failed, the loss of 20 or 30 ounces of blood, has procured profuse secretions.

*Chilliness.*—This symptom is usually accompanied by a small pulse, dry skin, load at the pit of the stomach, paleness of the countenance, giddiness in the head, and other symptoms which indicate a determination to internal organs. It is seated in the nerves of sensation, not at their sentient extremities, for it continues while the surface is burning hot to the touch, and the real heat of the system is several degrees above that of health. It is a false sensation of cold, and arises from some affection of the brain, or nerves of sensation at their origin. What this affection is, or what is the precise condition of the nerves, may be difficult to determine, as much as to determine what is the precise condition of the brain, or nerves which gives rise to that false sensation of pain which patients sometimes imagine they feel in amputated limbs. It is accompanied with symptoms of congestion of the brain and nervous system and abdominal viscera, and is relieved by bloodletting. This fact was exemplified in my own person, in a case of intermitting fever. I was freely bled at the commencement of the cold stage, which was removed in less than a minute. Reaction was light.

*Hurried expression of the countenance, difficulty of breathing and anxiety.*—These symptoms are owing to an affection of the respiratory system of nerves. The fourth pair of nerves, the par vagum, portio dura of the seventh pair, glosso-pharyngeal, and spinal accessory, arise from a separate and distinct column of medullary matter. These are all respiratory nerves, and unite the parts on which they are distributed, into sympathy with each other. (Charles Bell,) The fourth pair go to the eyes, and the portio dura to the face, and serve to bring these parts into sympathy with the chest. This may account for that peculiar expression of the countenance which accompanies hurried respiration during health. The par vagum, glosso pharyngeal and spinal accessory, which go to the muscles of the shoulder, chest, larynx, tongue, stomach and diaphragm, together with the portio dura of the seventh pair, and the patheticœ or fourth pair, are all found diseased after death. A network of turgid vessels envelopes their origin, and is connected with their investing membrane. This may also serve in some measure, to account for the loss of voice, which in some instances, cannot be raised above a whisper. While the same appearances at the root of the lingual nerve, may serve to account for the faltering of speech, and inability to put out the tongue.

*The load and tenderness in the epigastrium*, are evidently owing to congestion of the portal circle. Upon examination after death, all the viscera of the abdomen are found in a state of congestion.

*Burning sensation in the Stomach*—This symptom arises during the second or third stage, and succeeds the spasmodic pain of the first. It is accompanied by a red tongue, soreness of the throat, nausea and vomiting when any thing is taken into the stomach, sometimes appetite for food, and is succeeded by black vomit. It is owing to inflammation of the villous coat of the stomach, which inflammation *appears* to be excited by acrid secretions. Upon dissection inflammation of the stomach, and corrosion of its villous coat are observed. The organ is also found to contain the matter of black vomit.

*Black Vomit.*—Writers on Yellow Fever, have been fruitful enough in hypotheses, explanatory of the nature and origin of black vomit. Some have supposed it to be "an altered secretion of bile; others that it is "merely blood which has been effused from some of the small arteries, coagulated in the general cavity of the stomach, and its colour darkened by the absence of oxygen, or by some chemical decomposition;" others, again, that it is "a peculiar morbid secretion from the inflamed glands, and vessels of the stomach." The last hypothesis, seems to approach nearer the truth; for it cannot be denied, that it is a very *peculiar*, as well as a morbid secretion. With regard to the two first, I may briefly remark, that black vomit resembles in no respect either *bile* or coagulated blood; and, without attempting further to refute either of them, will add another to the number,—viz. That black vomit is a depraved secretion of the gastric juice. It is always found in the stomach, if any where; it is never seen in the liver or gall bladder; it is not always met with in the intestines; but is sometimes found there, in its progress through the canal. It is therefore reasonable to suppose, that it is a secretion from the internal surface of the stomach. But it would be very absurd and unphilosophical to suppose, that a new set of secretory organs has been called into existence, on this occasion. It must, therefore, be a depraved state of some of the *natural* secretions. Now the secretions of the stomach, during health, are mucous and the gastric juice. Although mucous secretions are met with in various parts of the body, and although they all become depraved during the disease, yet no matter resembling black vomit has been seen except in the stomach. Among the innumerable glands and secreting surfaces, none except those of the stomach are capable in health of secreting the gastric juice; or, in disease, of secreting that depraved fluid, called black vomit. That the secretion of gastric juice becomes depraved, as well as others, I think cannot be doubted. For among the general *wreck of functions*, why should this escape? It may become even more depraved, for the stomach suffers during the disease, more than any other organ which has a secreting surface.

In the last stage of the disease, and about the time when the secretion becomes so far depraved, as to assume the appearance of black vomit, a distressing sense of hunger comes on, and if the sense of burning in the stomach has not occurred before, it is felt now. Here the depraved secretion of gastric juice excites hunger, and by its acrid qualities inflames the stomach. The qualities of this *depraved secretion*, or black vomit, are such, as to destroy the coats of the stomach very soon; and the patient seems to die much sooner, than might be expected from the progressive increase of debility. I have seen the stomach of very recent subjects completely denuded of its internal coat, without much appearance of inflammation beneath.

*Spasms and Convulsions.*—These symptoms are usually preceded by a calm. In some instances, as I have said, nearly every symptom of disease subsides, and the patient believes himself restored. *A case.* Wm. Bishop, was brought into the charity Hospital, in the last stage of yellow fever. Twenty four hours before his death, without any sign which usually marks a favourable crisis, nearly every symptom subsided.

Skin and pulse became natural; no pain in the head or elsewhere remained; no sense of burning, or sickness at the stomach, or tenderness in the epigastrium; tongue clean and moist; walked about the ward; had an appetite for food, and believed himself well; when convulsions ensued and carried him off. Dissection in such cases exhibits collections of serum in the cavities of the brain, and congestions of the brain and spinal marrow. Similar appearances are observed in cases, in which the patient has died of idiopathick tetanus or convulsions. In the above case, the patient had not been bled.—*Query.* Might not active depletion, in the first stage, by relieving the congestion of the spinal marrow and the determination to the brain which caused the effusion, have prevented the fatal termination?

### TREATMENT.

The profession of medicine, has long been reproached, as hypothetical and uncertain; and it is a matter of astonishment to the candid observer, and not a little humiliating to professional pride, that opinions directly opposite, and modes of practice at direct variance with each other, although subject to the test of the same experience, should still divide the talent of the medical world. This can only arise from the readiness with which a portion of mankind adopt fashionable opinions, if sanctioned by the authority of great names, without stopping to enquire whether they are based upon sound philosophy and a corrrect estimate of facts. This kind of credulity tends more to retard the progress of sound pathology, than any other cause.

The period is anxiously to be wished for by every friend to science and humanity, when each member of the profession shall learn to exercise, on medical subjects, that faculty which gives man his distinctive prerogative over the rest of the animal creation. When the bed side, the dissecting room and the closet, shall unite their contributions, and every great subject be submitted to the scrutiny of reason and experience; then shall the science cease to be reproached as hypothetical, the dogmas of the schools give place to a more sound and rational pathology, and medicine attain that proud pre-eminence on the scale of science, which from its nature and usefulness it is destined to acquire.

The bugbears debility and putrescency, like the idle phantoms of a morbid fancy, still haunt the imagination of a portion of the faculty; and paralyse the arm which otherwise might be "strong to save." There is probably no opinion so pertinaciously adhered to, but is supported by so few facts or so little rational theory, as that of the fatal tendency of blood-letting in fevers. There is, it is true, something in the phenomena of fevers, which to a superficial observer may seem to indicate debility. But whoever will take the trouble to understand the laws and functions of the brain and nervous system, and trace the connection between the symptoms of the disease, and the appearances observed on dissection, will find more evidence of deranged action, than real debility.

It must not be supposed, that in fevers nor even in inflammations, the strength will be so great as in health. The vigour of the system is always the greatest when all its functions are carried on in the most perfect manner; and no sooner does disease take place, or, in other words, no sooner are the functions deranged, than appearances of debility occur. I have seen a patient with pneumonia, whose strength was so much prostrated in a few hours, that he could not bear an erect position, without fainting; yet several copious bleedings relieved the symptoms, increased the strength, and with the aid of other evacuants cured the patient. Every practitioner of much experience in diseases of a congestive nature or in severe inflammations, who has dared to use the lancet freely, must have witnessed its effects in restoring the strength and imparting vigour to the system. It would be folly to suppose that debility real, which is cured by remedies so powerfully debilitating to the system when in health. The following case will illustrate the effect of stimulants in such circumstances. M. Johnson (of Mississippi) had one of those forms of fever in which the patient suddenly falls down, as if from concussion of the brain.—Bloodletting speedily restored him to the use of his faculties. High arterial reaction soon succeeded which in a few days subsided. Debility supervened and stimulants were used. The debility increased rapidly under the stimulating course. The pulse became small and feeble, the skin, and, particularly the extremities were cold. He was unable to articulate, or swallow fluids. The respiration was slow, difficult, and attended with heaving of the chest. Coma supervened, and he was thought to be dying. Stimulants were now discontinued. He remained in the same situation an hour or two, when he had a copious evacuation by stool. The pulse, before scarcely perceptible at the wrist, immediately rose, and became full and soft, with a warm skin. There was an accession of strength, and his breathing became free and easy. He sat up in his bed, and conversed rationally. Was the debility in this case real, or only apparent? If real, why was it not relieved by stimulants, and why removed by catharsis? If, then, the debility is not real; but only apparent, it must be owing to a state of partial suspension or obstruction of the functions of the brain and nerves, which obstruction or suspension, depends upon a broken balance of the circulation, and too great a determination to those organs. The chief indications, therefore, evidently are to remove those local determinations and morbid accumulations, which obstruct the functions of the brain and nervous system, restore an equilibrium of the circulation, and save important organs from disorganization.

It must not be supposed, that bloodletting is indicated only in those cases in which it is necessary to subdue immoderate arterial reaction. Were this the case it would generally be unnecessary, for we have other remedies that will *certainly* produce that effect. It is required on other and more urgent accounts. It is necessary for breaking up local determinations, preserving the integrity of functions, and preventing disorganization. And, notwithstanding the prejudice which still exists in the minds of the timid or the ignorant against its use, it must form the sheet anchor of hope in this Herculean disease—at least as it prevails at this time. It is evident that in a disease of such a violent and rapid career, in which serious, and, not unfrequently irreparable injury to important organs occurs so early, and the period during which remedies may be applied with much prospect of success, is of such short duration, those which are the most prompt and certain in the operation, are chiefly to be relied on. We are possessed of no remedy of equal efficacy with bloodletting in breaking up local determinations; and consequently none so powerful in restoring the functions of the brain and nervous system, and of promoting an equilibrium of the circulation and excitability. By removing those determinations to the brain and nervous system, which obstruct their functions, the pain and weakness of the lower extremities are relieved; the secretions are promoted; the torpor of the bowels, and that torpor of the portal circle which causes the blood to accumulate there, are removed or greatly relieved; and, not least in point of importance, the danger of disorganization and effusion in the brain is greatly lessened.

If bloodletting be freely used, immediately upon the approach of febrile symptoms, it seldom fails to prevent the disease. If employed during the forming stage, and before important organs have suffered much from the disease, it invariably renders the case mild and manageable; and, if used at any time during the first, or early in the second stage of the complaint it cannot fail to mitigate the violence of the symptoms, and increase the prospect of recovery. In such cases, it relieves the headache, delirium, pain in the back, pain and sickness at the stomach, torpor of the bowels, promotes perspiration and the secretions generally. It also relieves the difficulty of breathing, load and tension of the epigastrium, and, instead of increasing the debility, tends to remove the lassitude and sense of fatigue.

Bloodletting may be carried to a great extent, if used during the first, or early in the second stage; but the approach of the third stage, should always be the signal for laying aside the lancet. For when it is established, there is no excessive reaction to subdue, and although the quantity of the circulating mass should be reduced, nature is to much enfeebled by the disease, to restore an equilibrium. Bloodletting under such circumstances will assuredly facilitate the approach of death. A good remedy is sometimes brought into discredit, by the timidity or ignorance of those who use it. It is not enough to know, that it is useful, but, it is necessary to understand the true principles of its application, as well as its *modus operandi*. It is, in deciding upon the time *when* and the manner *how*, that real talent unfolds itself; and distinguishes the man of science and skill from the blundering rotinist.—Bloodletting should be used *early* and *efficiently*, or not at all. I lately enquired of a practising physician, in this city, if he had found bloodletting useful in yellow fever, as it prevails at this time? He answered, that he had not. That he had used it in several cases, but the patients had all died. Astonished at a result so contrary to my own experience, and that of several other practitioners. I enquired how much they had been bled. He replied that in one instance (in which there seemed to have been more boldness displayed than in any other) the remedy had been carried to the *extent* of eight or ten ounces-—*Eight or ten ounces of blood* at a time from a *single patient* in yellow fever! And the patient did not die, instanter, of depletion? Surely this was a great stretch of human courage! The abstraction of eight or ten ounces of blood, can have but little effect in a disease of such violence as yellow fever. Such feeble and inert practice, can have but little effect, except to bring into discredit, the best remedy that ever graced the pages of medicine.

Dr. Cartwright of Natchez took sixty ounces of blood at a time, from a yellow fever patient, and cured the disease. Dr. Rush frequently bled to the extent of ninety ounces, during the progress of a single case, with the happiest effect. He often bled five or six times, and one case is related, in which the patient was bled fourteen times.

Dr. Johnson (Endemic of Bengal) who always bled "boldly and decisively, until the head and precordia were relieved"

observes, that were he to "go over the same ground again he would try a *still more active* course of depletion by bloodletting and purging." In the present endemic I have usually taken from 30 to 60 ounces. The blood does not invariably exhibit the inflammatory buff, even if bloodletting be necessary. The buffy appearance may always be observed, if the blood has been taken while there was an increased velocity of the circulation. If a person be bled while in health, the blood does not at first exhibit the buffy coat; but if the operation be repeated, often in a short space of time, a subsequent bleeding will exhibit that appearance. By lessening the quantity of the circulating mass, the velocity of the blood is greatly increased, so that as in cases where the inflammatory diathesis prevails, the blood passes more frequently through the lungs, is more completely decarbonized and consequently has a greater capacity for caloric, cools more slowly and thereby gives the fibrin more time to separate and appear on the surface, and thus it exhibits the inflammatory buff. Early in the first stage of fevers, the velocity of the circulation is not increased, but lessened, in consequence of the congestive state of the brain and nervous system, and reaction not having been established. Therefore, if blood be taken at this time, it will not exhibit the buffy coat, although bloodletting be essentially necessary. The absence of the buffy appearance, in the first stage, is owing to a languid state of the circulation, from the congestion. But in the last stage, the same circumstance arises from a languid state of the circulation from exhaustion. Therefore, as the absence of the buff in the first stage indicates the necessity of the lancet, so the appearance in the last stage, forbids its use.

*Cathartics.*—After bloodletting, no time should be lost in procuring free evacuations from the bowels. For this purpose, I have usually exhibited from 50 to 60 grs. of calomel and followed it up by Epsom salt, or castor oil, regulating the dose and repetition by the state of the stomach, together with frequent enemas. From the great torpor of the bowels, large doses of medicine are commonly necessary to procure evacuation. Cathartics usually procure great relief from all the symptoms, particularly if the stools are bilious. By promoting secretion from the bowels, they relieve the congestion of the portal circle. They also relieve the determination to the brain and nervous system; and thereby enable them to carry on the functions. Hence their influence in promoting secretions, from the liver, skin &c. of liberating the action of the heart and arteries, and of restoring warmth and activity to the surface and extremities. Frequently the physician is baffled in his efforts to procure catharsis by the exhibition of cathartics and enemas alone; in such cases, bloodletting, by relieving the congestive state of the brain and nerves, and particularly by its effect on the system of ganglionic nerves (which are always found inflamed after death) and the congestion of the portal circle, will powerfully assist our cathartic medicines. Upon the approach of the third stage, all copious evacuation should be carefully avoided.

*Cold affusion* is very powerful in subduing immoderate arterial reaction. It reduces the force and frequency of the pulse and relieves the pain in the head, back and extremities. A patient has been known to faint, from having a stream of cold water poured upon his head. It is also useful in promoting the secretions. But it is not so powerful in relieving congestions as bloodletting. It should be used as Dr. Currie has instructed us, only in those cases where the heat is steadily above the healthy standard, and there is no perspiration nor sense of chilliness present.

*Alteratives.*—In all fevers, but more especially in those of a malignant and inveterate character, the blood is hurried upon the brain and nervous system, obstructing or deranging the functions. The ganglionic system, which includes the secretory filaments for the abdominal viscera, as well as those which proceed more directly from the encephalon, are found diseased after death. This diseased state of the nerves, must necessarily derange the functions of the parts on which they are distributed. Hence the secretions from all the abdominal viscera are suppressed, increased in quantity or depraved in quality. In some instances, the secretion of bile is entirely suspended, none being found in the gall bladder after death:

At other times, it is increased in quantity, and depraved, consisting of a thin fluid or a thick dark coloured matter, different from healthy bile. The secretions from the bowels are also deranged. The stomach secretes an acrid matter, which corrodes and inflames its internal surface. The diseased state of the nerves also induces a torpor, and consequent congestion of the portal circle, which in its turn, serves to keep up the depraved, or suppressed state of the secretions. The skin likewise participates in this state of things. Its secretion is suppressed, or increased in quantity, depraved in quality and soon putrifies. Mercury has a *specific* action upon the glandular system, in promoting its various secretions when suppressed, and of correcting them when depraved. It infuses activity into that system. Those glands which are situated sufficiently superficial to be the subjects of observation, exhibit signs of increased action, such as tenderness and heat in their vicinity. If the surface and extremities were cold before, they become warm now, from the stimulus of the mercury upon their secretory organs. The blood circulates more freely to them. The brain and nervous system are consequently relieved, in some measure, of the congestions which obstruct their functions. The pulse becomes full and strong, and healthy secretions commence from the salivary glands, the skin, the liver, all the abdominal viscera. These evacuations serve further, to deplete and relieve congestions, while the activity diffused into every part of the glandular system, serves to promote that equilibrium in the circulation and excitability, in which health consists. But in some cases, the congestion of the brain and nervous system, so completely obstructs their functions, and renders the glandular system so torpid, that mercury will not procure secretions; but causes inflammation, ulceration, gangrene and sloughing from the mouth and throat. In such cases "pushing the mercurial treatment" without the proper preparatory measures, may do incalculable mischief by the irritation it causes. Bloodletting, cold affusions, cathartics, and emetics, when there is nothing to forbid their use, by relieving the determination to the brain and nervous system, serve to assist the calomel in restoring the secretions. Unfortunately, too often the difficulty of procuring secretion is evident, only, in that stage in which bloodletting and other active evacuants, are inadmissible. The first and second stages, having passed by while the physician still had hopes of success; he discovers his mistake, when it is too late to apply an efficient remedy. This obstinate condition may generally be apprehended, however, when there are strong signs of determination to the brain and nervous system, or of great congestion of the viscera. In such cases, depletion is necessary to enable medicines to have their proper effect; and to save the organs which suffer most, from disorganization. In general, if active depletion be used early in the disease, very little need be apprehended from torpor of the glands, and calomel will seldom fail to procure secretion. In nearly every case that came under my observation, in which the patient was freely bled and purged early in the disease, calomel acted readily enough upon the glands.

*Stimulants.*—Those congestions which occur early in the disease, do not depend, primarily, upon a diminution of the force of the heart and arteries, but upon a broken balance of the circulation; whereby determinations to the brain and nervous system take place, and obstruct their functions. These primary congestions are, therefore, of a *sthenic* character, and frequently continue to the very close of the disease. In such cases the exhibition of internal stimulants gives no relief, but tends further to depress the pulse and strength. On the contrary, the patient always obtains relief from evacuants. Even when the debility is so great, and the disease so far advanced, that no rational hopes can be entertained of recovery, cathartics procure temporary relief, and an accession of strength. But not unfrequently, upon the approach of the third stage the congestions change their character; the force of the determinations which caused them, in the first stage, having been lessened, they depend for the most part upon debility of the arterial system, and the functions of the brain and nervous system having been impaired by previous local determinations. These are, therefore, *asthenic* congestions, and may be treated with stimulants both local and general. The increased fullness and regularity of the pulse, soft perspirable state of the skin, warmth of the extremities, returning consciousness and reason, refreshing sleep, increase of strength, and the general improvement of the symptoms under its use, will show the propriety of its continuance; while on the contrary, a depressed pulse, stupor, delirium, dryness of the skin, restlessness, &c. supervening, should be the signal for laying them aside.

I forbear to say any thing relative to the various auxiliary remedies, usually adopted in the treatment of yellow fever; having nothing new to offer relative to them, and every practitioner knowing how to estimate their value.

